# Myocardial injury is a risk factor for 6-week mortality in liver cirrhosis associated esophagogastric variceal bleeding

**DOI:** 10.1038/s41598-023-33325-6

**Published:** 2023-04-17

**Authors:** Bihan Liu, Qi Li, Huiguo Ding, Shanshan Wang, Lifang Pang, Lei Li

**Affiliations:** 1 Department of Gastroenterology and Hepatology, Beijing You’an Hospital, Capital Medical University, Beijing, 100069 China; 2 Department of Molecular Biology, Beijing Institute of Hepatology, Beijing You’an Hospital, Capital Medical University, Beijing, 100069 China; 3 Department of Electrocardiography, Beijing You’an Hospital, Capital Medical University, Beijing, 100069 China

**Keywords:** Diseases, Gastroenterology, Medical research, Risk factors, Signs and symptoms

## Abstract

This study sought to investigate risk factors for 6-week mortality of patients with decompensated liver cirrhosis associated esophagogastric variceal bleeding (EGVB) and clinical characteristics of myocardial injury in cirrhotic patients with EGVB. This retrospective cohort study included 249 patients with decompensated liver cirrhosis associated EGVB in the Department of Emergency. Patients were divided into two groups including liver cirrhosis associated EGVB without myocardial injury and liver cirrhosis associated EGVB with myocardial injury. Myocardial injury, recurrent bleeding, total bilirubin (TBIL) level and dyslipidemia are independent risk factors for 6-week mortality in liver cirrhosis associated EGVB. Among all patients with liver cirrhosis associated EGVB, 90 (36.2%) had myocardial injury and 159 individuals (63.8%) not. The 6-week mortality in the group with myocardial injury was 21%, which was significantly higher than that of 7% in the group without myocardial injury. More patients in the myocardial injury group smoked, had moderate to severe esophageal varices, liver failure, and Child–Pugh C liver function compared to the non-myocardial injury group. Myocardial injury, recurrent bleeding, TBIL level and dyslipidemia are independent risk factors for death within 6 weeks in liver cirrhosis associated EGVB. The 6-week mortality is considerably higher in patients with myocardial injury in liver cirrhosis associated EGVB than those without myocardial injury.

## Introduction

In liver cirrhosis, esophagogastric variceal bleeding (EGVB) is a potentially fatal complication, which is responsible for 70% of all acute upper gastrointestinal bleeding (AUGIB)^1–3^. In severe cases, EGVB may result in deadly hemorrhagic shock with significant blood loss, which leads to a sharp increase in short-term mortality^4^. In acute EGVB, the 6-week mortality rate is between 15 and 20%^5^.

10–25% of patients with severe AUGIB develop myocardial ischemia or acute myocardial infarction (AMI) with a 6-week mortality rate of 15–20%^6,7^. The mortality rate of AUGIB with myocardial injury, particularly myocardial infarction, increases significantly. Cappell MS reported that patients with AUGIB and myocardial injury had a significantly higher mortality rate than controls (33 vs 8%)^8^. Age, combined underlying diseases, ST-T elevation, and cTn elevation are found to be independent risk factors for myocardial injury when combined with AUGIB^9^. The mechanism of gastrointestinal bleeding-induced myocardial injury is complicated. It could be due to gastrointestinal bleeding and insufficient coronary perfusion, which causes myocardial ischemia and accelerates myocardial damage^7,10^. Cirrhosis-related structural and functional abnormalities of the liver lead to portal hypertension. Hyperdynamic circulation is caused by the activation of the renin–angiotensin–aldosterone system (RAAS), sympathetic nervous system (SNS), and the interaction of cytokines and inflammatory factors^11,12^. Long-term hemodynamic changes, as well as structural and functional heart changes, all contribute to myocardial injury in liver cirrhosis^13^.

At present, there are few studies on myocardial injury in patients with liver cirrhosis associated EGVB. It remains unclear whether myocardial injury influences the prognosis of patients with liver cirrhosis associated EGVB. Therefore, with a retrospective cohort, we investigated risk factors of 6-week mortality in liver cirrhosis associated EGVB in our hospital and analyzed the clinical characteristics of liver cirrhosis associated EGVB combined with myocardial injury.

## Methods

### Data source

We conducted a single-center retrospective cohort study to assess risk factors for 6-week mortality in patients with decompensated liver cirrhosis associated EGVB. From April 1st to September 30th, 2021, 461 patients with liver cirrhosis and AUGIB were admitted to the Department of Emergency at Beijing You’an Hospital, Capital Medical University. Finally, 249 patients with esophagogastric variceal bleeding confirmed by endoscopy were enrolled in this study. Patients were divided into two groups including patients with myocardial injury and those without. In this study, for patients with multiple AUGIB admissions, only the clinical data from the first admission were analyzed.

Patients were monitored for 6 weeks. The primary endpoint is death in 6 weeks. If the patients were discharged, their prognosis was recorded via a phone call from their relatives. The Ethics Committee of Beijing You’an Hospital, Capital Medical University approved the protocols of the study, and written informed consent was waived due to the retrospective nature of this study.

### Diagnostic criteria

Diagnosis of decompensated liver cirrhosis is based on liver dysfunction, portal hypertension and complications including variceal bleeding, ascites, hepatic encephalopathy and etc.

AUGIB is characterized by tarry stools, coffee-colored vomitus, bloody vomiting, or anemia-related symptoms.

Endoscopy provides evidence of EVGB. Esophagogastric varices are classified as mild, moderate and severe depending on the morphology of the varices, the presence or absence of red signs and the risk of bleeding as the following. Mild (G1): straight or slightly tortuous GOV with no red signs. Moderate (G2): straight or slightly tortuous GOV with red signs or serpentine tortuous GOV with no red signs. Severe (G3): serpentine tortuous GOV with red signs, or beaded, nodular or warty GOV (with or without red signs)^14^.

All enrolled patients underwent ECG examination as well as cardiac enzyme examination. Based on relevant diagnostic guidelines, myocardial injury is defined as as TnI > 0.056 ng/L and common ST-T changes on the ECG (abnormal changes in the ECG)^9,15^. The normal ST-T segment is mostly an isoelectric line. In normal condition, the downward shift of the ST segment does not exceed 0.05 mV in either lead and the upward shift of the ST segment does not exceed 0.3 mV in leads V1, V2, 0.5 mV in lead V3 and 0.1 mV in leads V4–V6. ST-T changes are defined as ST-segment depression, i.e. a downward shift of the ST-segment greater than 0.05 mV, and T-wave depression, i.e. T-wave amplitude less than 1/10th of the R-wave in the same lead, bimodal or inverted changes^15,16^.

### Inclusion criteria and exclusion criteria

Patients over 18 years old with a confirmed diagnosis of decompensated liver cirrhosis, and endoscopic evidence of esophagogastric fundic varices within one month when admitted to the Department of Emergency were enrolled in this study.

Patients with the following conditions were excluded from the study: (1) patients without liver cirrhosis, (2) patients without endoscopic findings or without esophageal and gastric fundus varices, (3) patients bleeding from sources other than ruptured esophagogastric fundic varices, and (4) patients without complete clinical data.

### Information collection

General clinical data including age, gender, symptoms (melena, hematemesis, hematochezia), systolic blood pressure, diastolic blood pressure, mean arterial pressure, personal history (smoking, alcohol), cause of liver cirrhosis (Child–Pugh score), underlying diseases (atrial fibrillation, hypertension, diabetes, chronic kidney disease, liver failure, liver cancer, other malignancies and etc.), complications (hepatic encephalopathy, ascites, portal vein thrombosis, esophageal varices), laboratory tests (blood routine, biochemical, coagulation, myocardial enzymes), gastroscopy within 1 month of admission, first bleeding, endoscopic treatment, length of hospitalization, ICU, 6-week death, death reason were collected.

### Statistical analysis

Measurement data with a normally distribution were reported as mean standard deviation (xs) and examined using the independent sample *t*-test using statistical software SPSS 22.0. The non-parametric rank-sum test was used to assess the non-normally distributed measurement data, which was expressed as the median (interquartile spacing). The chi-square test was used to examine count data (composition ratio) using examples. For multivariate analysis, logistic regression included variables with substantial differences in univariate analysis. It is statistically significant if *P* < 0.05.

### Ethical approval and consent to participate

This study was approved and consented to by the Ethics Committee of Beijing You’an Hospital, Capital Medical University. This study was approved and consented by the Ethics Committee of Beijing You’an Hospital, Capital Medical University, in accordance with the Declaration of Helsinki. The data used in this study were anonymous.

## Results

### Characteristics of patients

**Figure 1:**
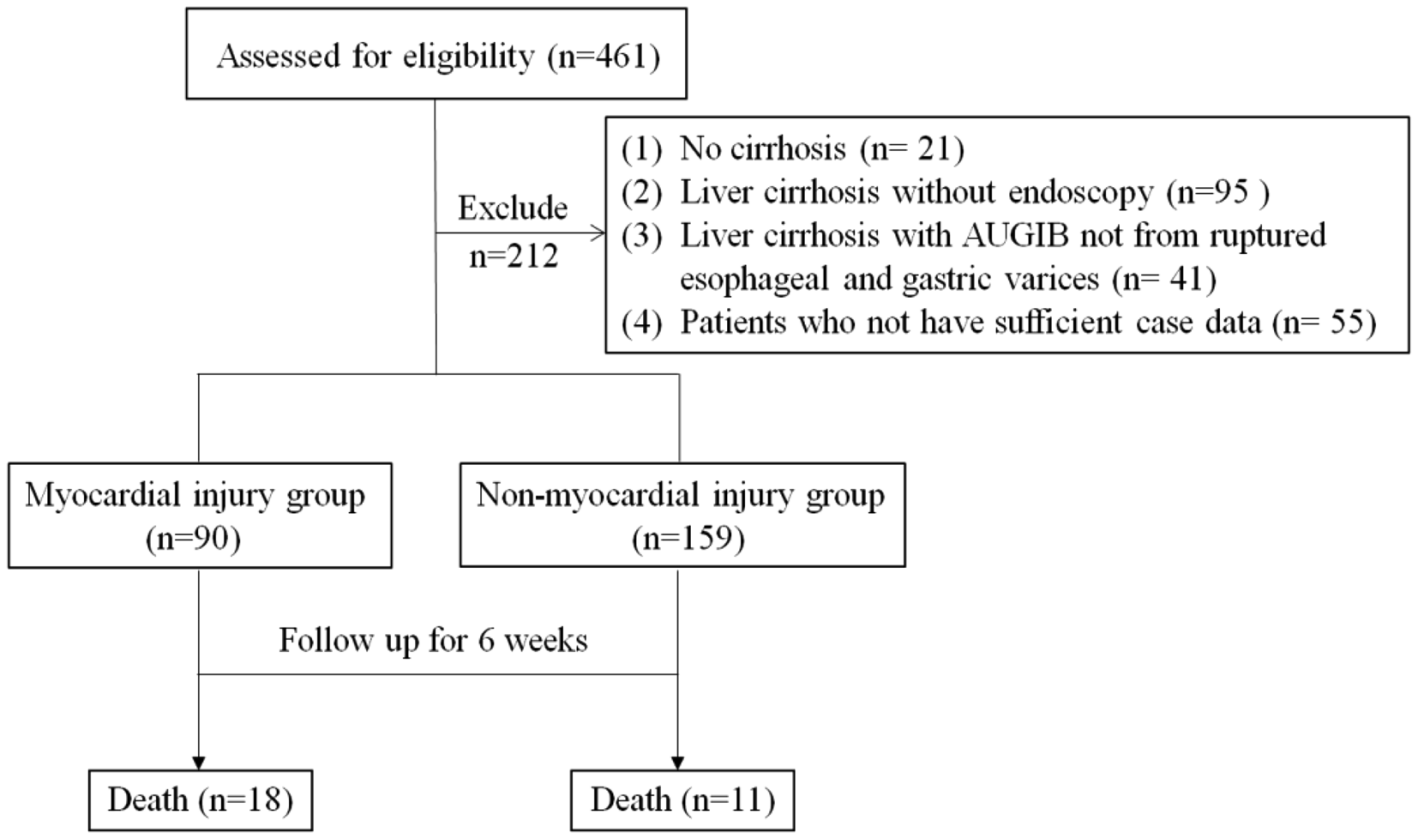
Flow chart for patient selection.

461 patients with decompensated liver cirrhosis were assessed for eligibility, and 249 patients who fulfilled the inclusion criteria were finally enrolled in this study. The mean age of the patients was 58.10 ± 11.14 years. Among them, 182 (73.09%) were male and 67 (26.91%) were female. All 249 patients received endoscopy, which demonstrated that 34 (13.65%) patients had mild esophago-gastric varices, 57 (22.89%) moderate esophago-gastric varices and 154 (61.85%) severe esophago-gastric varices. Among them, 90 (36.14%) patients were diagnosed with myocardial injury, and 159 (63.8%) patients without myocardial injury. Twenty-nine patients died in 6 weeks, representing 11.65% of all patients included in the study (Fig. 1).

### Risk factors for 6-week mortality in liver cirrhosis associated EGVB

There were no statistically significant differences between patients who died within 6 weeks and those who survived in terms of gender, systolic blood pressure, diastolic blood pressure, Hematemesis, Hb, Hct, PLT, BUN, Cr, ALT, AST, TBIL, INR, Albumin, PT, APTT, smoking, alcohol consumption, endoscopic treatment and length of hospital stay. However, more patients who died within 6 weeks had comorbid underlying conditions including hypertension, diabetes, heart failure, myocardial injury, dyslipidemia, portal vein thrombosis and liver cancer than those who survived within 6 weeks. Patients who died within 6 weeks tended to experience recurrent bleeding, be older, be admitted to ICU, and have higher TBIL level (Table 1).

**Table 1:** Characteristics of patients in term of death within 6 weeks.

	Death within 6 weeks (n = 29)	Survivor within 6 weeks (n = 220)	P value
Sex (male/female)	24 (0.828)/5(0.172)	158(0.718)/62(0.282)	0.268
Age	59.2 ± 8.3	57.9 ± 11.5	0.038
Melena	14 (0.48)	125 (0.57)	0.52
Hematemesis	7 (0.24)	163 (0.74)	0.52
Systolic blood pressure	112.6 ± 22.9	114.2 ± 21	0.5
Diastolic blood pressure	66.6 ± 13.9	55.2 ± 7.1	0.49
Hypertension	11 (0.40)	45 (0.20)	0.034
Diabetes	14 (0.48)	58 (0.26)	0.015
Smoking	11 (0.38)	96 (0.44)	0.353
Alcohol consumption	16 (0.55)	116 (0.53)	0.481
Liver cancer	11 (0.40)	56 (0.25)	0.02
Liver failure	2 (0.07)	26 (0.12)	0.337
Coronary heart disease	7 (0.24)	15 (0.07)	0.101
Dyslipidemia	8 (0.27)	25 (0.11)	0.023
Child–Pugh			0.26
A	2 (0.068)	24 (0.11)	
B	10 (0.35)	103 (0.47)	
C	17 (0.59)	94 (0.43)	
Portal vein thrombosis	16 (0.55)	7 (0.35)	0.032
Heart failure	8 (0.27)	6 (0.03)	< 0.001
Myocardial injury	18 (0.62)	11 (0.05)	< 0.001
Endoscopic treatment	14 (0.48)	67 (0.30)	0.46
Length of hospitalization	11.4 ± 6.4	14.8 ± 7.5	0.331
ICU	6 (0.21)	18 (0.08)	0.032
Recurrent bleeding	11(0.38)	34 (0.15)	0.003
Hb (g/L)	78.4 ± 24.1	77.8 ± 24.1	0.848
Hct	24.2 ± 7.4	23.7 ± 6.8	0.547
PLT	100.2 ± 54.4	96.6 ± 53.3	0.39
BUN	10.5 ± 5.7	11.0 ± 5.6	0.787
Cr	80.4 ± 48.6	70.9 ± 40.3	0.953
ALT	47.6 ± 56.3	62.6 ± 225.1	0.43
AST	124.6 ± 254.9	96.7 ± 314.7	0.577
TBIL	58.5 ± 82.0	34.9 ± 40.5	< 0.001
INR	1.51 ± 0.5	1.5 ± 0.4	0.973
Albumin (g/L)	34.7 ± 32.9	29.0 ± 5.4	< 0.001
PT	16.9 ± 5.0	16.8 ± 4.7	0.975
APTT	31.5 ± 6.0	32.5 ± 10.6	0.718
INR	1.51 ± 0.5	1.5 ± 0.4	0.973

A multifactorial logistic regression analysis was performed on the variables that were statistically significant for 6-week mortality in liver cirrhosis associated EGVB. We found that myocardial injury, TBIL level, dyslipidemia and recurrent bleeding were independent risk factors for 6-week mortality in liver cirrhosis associated EGVB (Table 2).

**Table 2:** Multivariable analysis for 6-week mortality in cirrhosis associated EGVB.

	B	S.E.	Wald	df	Sig	Exp (B)
Age	0.010	0.024	0.169	1	0.681	1.010
Hypertension	− 0.173	0.692	0.062	1	0.803	0.841
Diabetes	0.073	0.563	0.017	1	0.897	1.076
Liver cancer	− 0.010	0.555	0.000	1	0.985	0.990
Coronary heart disease	0.381	0.719	0.281	1	0.596	1.464
Heart failure	− 1.193	0.970	0.000	1	1.000	0.000
dyslipidemia	2.676	0.814	10.817	1	0.001	14.527
Portal vein thrombosis	0.462	0.499	0.859	1	0.354	1.587
Recurrent bleeding	1.280	0.516	6.146	1	0.013	3.597
ICU	0.245	0.861	0.081	1	0.775	1.278
Myocardial injury	1.105	0.517	4.558	1	0.033	3.019
TBIL	0.008	0.004	4.727	1	0.030	1.008
Albumin (g/L)	0.027	0.040	0.463	1	0.496	1.028

Of all patients, 29 died within 6 weeks, 45 patients experienced rebleeding, of which 11 died within 6 weeks. The rate of recurrent bleeding among patients who died within 6 weeks was 38%, significantly higher than that of recurrent bleeding among patients who survived in 6 weeks. Of the 29 patients who died within 6 weeks, 8 died of heart failure due to myocardial injury and 5 died of myocardial infarction due to myocardial injury, 11 (38%) died from bleeding due to EGVB recurrence, 3 (10.34%) from shock due to abdominal infection, 1 (3.45%) from acute and chronic liver failure (ACLF), and 1 (3.45%) from primary liver cancer. In terms of methods of haemostasis, of the 45 patients who re-bleed, five of the 11 (45%) who died within 6 weeks were treated with esophageal sclerotherapy in the emergency department, 3 (27%) were treated with esophageal sclerotherapy and gastric tissue glue in the ward, and a further 3 (27%) died at home from recurrent gastrointestinal haemorrhage. Of those who survived within 6 weeks, 16 were stopped by esophageal sclerotherapy at the time of recurrent bleeding, 15 by esophageal sclerotherapy with gastric tissue glue and 2 by endoscopic treatment in the emergency department.

### Features of patients in the myocardial injury group and non-myocardial injury group

There were no statistically significant differences in sex, age, symptoms, systolic blood pressure, diastolic blood pressure, hepatic encephalopathy, alcohol consumption, coronary heart disease, dyslipidemia, and combined underlying diseases including atrial fibrillation, diabetes mellitus, chronic kidney disease, end-stage renal disease, liver cancer, other malignancies and portal vein thrombosis between the myocardial injury group and the non-myocardial injury group. However, more patients with myocardial injury smoked, had moderate or severe esophageal varices, liver failure and liver function of Child–Pugh C compared with those without myocardial injury. Furthermore, patients in the myocardial injury group had lower mean arterial pressure than those in the non-myocardial injury group (Table 3).

**Table 3:** Baseline characteristics of patients in the myocardial injury group and non-myocardial injury group.

Characteristics	Myocardial injury (n = 90)	Non-myocardial injury (n = 159)	*P* value
Sex	72 (0.8)	110 (0.69)	0.064
Age (years)	57.9 ± 12.0	58.2 ± 10.7	0.131
Melena	52 (0.58)	87 (0.55)	0.640
Hematemesis	72 (0.8)	113 (0.71)	0.121
Hematochezia	10 (0.11)	22 (0.14)	0.537
Systolic blood pressure	109.7 ± 20.1	116.5 ± 21.8	0.073
Diastolic blood pressure	62.8 ± 14.0	67.3 ± 13.6	0.632
Mean arterial pressure (mmHg)	18.0 ± 15.6	88.2 ± 16.4	0.014
Hepatic encephalopathy			0.061
None	29 (0.32)	73 (0.46)	
Stage 1–2	60 (0.67)	85 (0.46)	
Stage 3–4	1 (0.01)	1 (0.01)	
Smoking	35 (0.39)	12 (0.08)	< 0.001
Alcohol consumption	47 (0.52)	85 (0.53)	0.851
Hypertension	15 (0.17)	34 (0.21)	0.368
Diabetes	20 (0.22)	50 (0.31)	0.121
Coronary heart disease	9 (0.1)	13 (0.08)	0.393
Dyslipidemia	9 (0.1)	24 (0.15)	0.173
Atrial fibrillation	5 (0.05)	4 (0.03)	0.217
Chronic kidney disease	7 (0.08)	7 (0.04)	0.267
End-stage renal disease	2 (0.02)	1 (0.01)	0.365
Liver cancer	30 (0.33)	37 (0.23)	0.085
Other malignancies	3 (0.03)	6 (0.04)	0.858
Liver failure	16 (0.18)	12 (0.08)	0.014
Portal vein thrombosis	37 (0.41)	56 (0.35)	0.356
Esophageal varices			0.032
Mild	7 (0.07)	28 (0.18)	
Moderate or severe	83 (0.92)	131 (0.82)	
Child–Pugh			< 0.001
A	3 (0.03)	23 (0.14)	
B	29 (0.32)	83 (0.52)	
C	57 (0.63)	52 (0.33)	

### Laboratory examination of patients in the myocardial injury group and non-myocardial injury group

Compared to patients without myocardial injury, patients with myocardial injury had significantly higher level of ALT, AST, TBIL, INR and PT. We failed to observe significant differences in other values at baseline including Hb, Hct, PLT, BUN, Albumin, APTT and Cr between the two groups (Table 4).

**Table 4:** Laboratory examination of patients in the myocardial injury group and non-myocardial injury group.

Characteristics	Myocardial injury (n = 90)	Non-myocardial injury (n = 159)	*P* value
Hb (g/L)	74.1 ± 24.5	80.0 ± 23.8	0.586
Hct (g/L)	22.6 ± 7.0	24.5 ± 6.8	0.526
PLT (10^9^/L)	99.1 ± 53.4	95.8 ± 58.9	0.683
BUN (mmol/L)	11.5 ± 6.2	10.6 ± 5.2	0.17
Cr (μmol/L)	79.2 ± 40.1	67.9 ± 40.2	0.093
ALT (U/L)	108.0 ± 346.5	33.9 ± 29.7	< 0.001
AST (U/L)	181.5 ± 491.5	53.6 ± 74.0	< 0.001
TBIL (μmol/L)	46.6 ± 55.7	34.2 ± 42.0	0.036
INR	1.65 ± 0.48	1.42 ± 0.36	0.021
Albumin (g/L)	28.6 ± 19.3	30.2 ± 5.1	0.127
PT	18.4 ± 5.3	15.8 ± 4.0	0.023
APTT	33.1 ± 7.3	32.0 ± 11.7	0.909

### Treatment and prognosis of patients in the myocardial injury group and non-myocardial injury group

As shown in Table 5, the 6-week mortality rate was 21% in the myocardial injury group, which was significantly higher than 7% in the non-myocardial injury group. Other indexes such as first bleeding, endoscopic treatment and admission to the ICU seemed to occur more in the non-myocardial injury group than the myocardial injury group, while no statistical significance was found. Patients with myocardial injury held longer length of hospitalization than those without myocardial injury, but there was no marked difference, either.

**Table 5:** Treatment and prognosis of patients in the myocardial injury group and non-myocardial injury group.

Characteristics	Myocardial injury (n = 90)	Non-myocardial injury (n = 159)	*P* value
First bleeding	37 (0.41)	64 (0.40)	0.894
Endoscopic treatment	54 (0.60)	104 (0.65)	0.395
Length of hospitalization	14.8 ± 5.1	14.4 ± 6.0	0.096
ICU	9 (0.10)	13 (0.08)	0.438
6-week mortality	18 (0.21)	11 (0.07)	0.002

## Discussion

AUGIB due to esophageal varices in liver cirrhosis is a relatively common clinical emergency, often accompanied by cerebrovascular accidents, heart failure, chronic renal insufficiency, etc. Recent studies have reported that AUGIB without esophageal varices is likely to be complicated by myocardial injury and the occurrence of AUGIB often masks the potential myocardial injury. In severe cases, it can even be complicated by acute myocardial infarction, which is life-threatening^6^. In this study, we found that the 6-week mortality rate for cirrhosis combined with EGVB was 11.6%, the incidence of myocardial injury in cirrhosis combined with EGVB was 36%. Myocardial injury, recurrent bleeding, dyslipidemia and TBIL were independent risk factors for death within 6 weeks in cirrhosis with EGVB.

Statistically, recurrent bleeding usually occurs in moderate to severe oesophageal varices, and these patients have a high probability of recurrent bleeding even after the first complete hemostasis, which can easily lead to the development of shock and consequently death in the short term. The results of a retrospective population-based case–control study at the National University Hospital of Iceland showed that in 5-year follow-up, patients with AUGIB had six times the risk of rebleeding compared to controls for bleeding events. Even after correcting for age and comorbidities, short-term and 5-year mortality rates were significantly higher in AUGIB patients than in controls, which is consistent with our results ^17^. In two studies from Finland, where patients with AUGIB^18^ were followed-up for one year, the rebleeding rate was 11 and 14%, respectively. These studies have shown that patients with rebleeding tend to have higher mortality rate than those without rebleeding. This is consistent with our results.

As we know, our study is the first to find that myocardial injury is an independent risk factors for death within 6 weeks in liver cirrhosis associated EGVB. In our cohort, 36% of the patients had myocardial injury, which was similar to I-Chen Wu’s data^14^. They reported that the incidence of mild myocardial injury in patients with AUGIB was about 7.74%, and the moderate myocardial injury rate was 32.9%^9^. Iser et al. conducted a retrospective study in Australia including 156 patients and found that the incidence of combined myocardial injury in patients with AUGIB was 19%^19^. The significant variation in the incidence of myocardial injury between these studies may lie in different diagnostic definition for myocardial injury and the criteria for patients’ inclusion. I-Chen Wu et al.^9^ concluded that patients with AUGIB who had a history of cirrhosis and three or more risk factors for heart disease were at a significantly higher risk of myocardial injury. We found that the 6-week mortality in the group with myocardial injury was 21%, which was significantly higher than that of 7% in the group without myocardial injury. However, Cappell MS reported that patients with AUGIB and myocardial injury had a significantly higher mortality rate than controls (33 vs 8%)^8^. Furthermore, we found that coronary heart disease are not prognostic factors for death within 6 weeks in liver cirrhosis associated EGVB. Coronary heart disease are a widespread phenomenon associated with different clinical entities. It is characterized by the formation of arterial plaques that narrow the lumen of the coronary arteries, leading to episodes of angina pectoris or persistent angina^20^. In our study, there are 22 patients with a past history of coronary heart disease, some with stable coronary artery disease and some with acute coronary syndrome with stenting. However, these patients with coronary heart disease may not present with typical symptoms during the process of gastrointestinal bleeding. Myocardial injury is an acute process that is not directly related to a history of previous coronary artery disease. In addition, it is also possible that our total sample is not large enough and should be further validated in the future on the basis of an expanded sample.

Our study also found that dyslipidemia and TBIL were independent risk factors for death within 6 weeks in cirrhosis with EGVB. Dyslipidemia usually refers to elevated serum levels of cholesterol, triglycerides, and LDL cholesterol, and lower HDL cholesterol levels^21^. Dyslipidemia can lead to atherosclerotic cardiovascular disease such as coronary heart disease, as well as increase the risk of death^22^. Also, dyslipidemia is an important factor contributing to increased mortality from cirrhosis, with hazard ratios ranging from 2.1 to 8.9^23,24^ and odds ratios from 6.6 to 26.9% reported in different studies^24,25^. A previous study on myocardial injury in COVID-19 patients found that preexisting cardiovascular and metabolic comorbidities, as well as abnormal levels of metabolic markers, were more frequently observed in COVID-19 patients with myocardial injury^26^. These results suggest that patients with abnormal lipid metabolism, are at increased risk of myocardial injury and death, which is accordance with our results. The TBIL level can be used to assess the degree of jaundice and hepatobiliary damage, which is associated with immune dysfunction, increased bacterial translocation, and deterioration of nutritional status and liver function^27^. Many prognostic studies have reported that TBIL is an important indicator of survival in patients with liver diseases^28^. Hyperbilirubinemia is considered to be a potential high-risk factor associated with postoperative mortality in hilar cholangiocarcinoma^29^. A recent retrospective, single-center study once again confirmed that preoperative bilirubin concentrations were an important risk factor for postoperative severe complications and mortality in perihilar cholangiocarcinoma^30^.

The possible mechanism of myocardial injury complicated by AUGIB is inadequate coronary artery perfusion due to reduced effective circulating blood volume. In addition, due to massive blood loss, hemoglobin carrying oxygen is reduced, which further decreases the oxygen supply capacity of the heart^31^. The decrease in circulating blood volume reflexively activates the sympathetic nervous system and increases the release of angiotensin and catecholamines, which stimulate strong constriction of the coronary arteries, causing detachment or even rupture of unstable plaques in the coronary arteries, leading to acute myocardial infarction. In addition, the activation of the coagulation system leads to the formation of blood clots, which in turn induces myocardial ischemia, etc.^10^.

As for myocardial injury in cirrhosis associated EGVB, the mechanism remains largely unclear. High output heart failure occurs in patients with liver cirrhosis, which is a syndrome known as cirrhotic cardiomyopathy (CCM). When patients with liver cirrhosis are challenged by medications or physiological stress, they exhibit low ventricular reactivity, which may exacerbate (subclinical) heart failure and myocardial injury^32^. Several studies in recent years have shown a progressive deterioration of cardiac function during cirrhosis, manifested by heart failure and reduced cardiac output, leading to loss of hyperdynamic circulation. Other findings regarding this particular cardiomyopathy include impaired systolic response and reduced adequate arterial blood volume^33^ and its positive correlation with the degree of liver failure^34,35^.

In this study, we found that patients with underlying diseases such as hypertension, diabetes mellitus, heart failure, and liver cancer have significantly higher mortality rates within 6 weeks. When hypertensive patients do not take active and effective antihypertensive treatment, they are prone to develop hypertensive encephalopathy, hypertensive crisis, hypertensive cardiac insufficiency and cerebral hemorrhage, which can cause cerebral artery rupture, hemorrhage or occlusion, which can lead to stroke resulting in death^36^. People with diabetes are highly susceptible to cardiovascular complications, and exposure to cardiovascular risk factors can exacerbate the progression of the disease and lead to increased mortality. The occurrence of heart failure undoubtedly increases the risk of death in patients. Patients with heart failure are prone to malignant arrhythmias, which cause severe ischemia and hypoxia to occur due to a drastic reduction in effective pumping of the heart, leading to severe hypoxemia, respiratory failure, and eventually progressing to multi-organ failure, which can cause death^37^.

We also analyzed systolic blood pressure, diastolic blood pressure values, and mean arterial pressure in patients with EGVB associated with liver cirrhosis at admission. We found that systolic and diastolic blood pressures were not statistically different between the myocardial and non-myocardial injury groups. In contrast, mean arterial pressure (*P* = 0.014) was significantly different between the two groups. Systolic blood pressure mainly reflects cardiac contractility and displacement, while diastolic blood pressure reflects peripheral vascular resistance. Mean arterial blood pressure is the average propulsive force given by the heart to blood in the arteries throughout the cardiac cycle and indicates the pressure that drives blood flow during cardiac contraction and ejection^38^. When AUGIB occurs, blood volume decreases dramatically, and blood supply to the peripheral circulation is increased early by enhancing myocardial contractility and increasing cardiac output when systolic blood pressure rises to some extent. On the other hand, diastolic blood pressure reflects the degree of vascular elasticity and may decrease during this period. It has been found that mean arterial pressure of ≤ 65 mmHg increases the risk of cardiovascular disease and death^39^. Some investigators believe that coronary artery perfusion is inadequate when mean arterial pressure decreases substantially, leading to an increased risk of cardiovascular events and death^40^. Our study also confirmed the significant difference in mean arterial pressure between the two groups.

In addition, there are clinical evidences of an association between the degree of hepatic insufficiency and the severity of cardiomyopathy due to cirrhosis. A recent study on advanced cirrhosis pointed out an association between the degree of CCM and Model for End-Stage Liver Disease (MELD) scores^41,42^. Hepatocellular injury and elevated levels of various serological markers such as ALT, AST, TNF-α, YKL-40 (related to liver injury and inflammation) exacerbate hemodynamic disturbances and hyperdynamic circulation which further aggravate myocardial injury^13,35,43^. It was concluded that the degree of liver failure was positively correlated with myocardial injury in patients with cirrhosis^44,45^. This coincides with our results, in which liver failure and level of ALT were significantly different between the myocardial and non-myocardial injury groups. The liver plays a central role in the blood clotting process. Coagulation disorders are prevalent in patients with acute and chronic liver disease, leading to increased morbidity and mortality in patients with liver diseases^46^. Our study showed coagulation indexes including international standard (INR) and prothrombin time (PT) were significantly higher in patients with myocardial injury than those without myocardial injury. Thrombin is a multifunctional protease that promotes coagulation, inflammation, and apoptosis^47^. Recent evidence suggests that thrombin-activated protease-activated receptor-1 regulates cardiomyocyte apoptosis^48^. High concentrations of thrombin have a pro-apoptotic effect on cultured vascular smooth muscle cells^49^, and thrombin has a direct potential detrimental effect on cardiomyocytes.

The advantages of our research include that our hospital is a specialized liver disease hospital, which holds a well-judged EGVB cohort of cirrhosis with strict inclusion criteria, exclusion criteria, availability of standardized results evaluation. However, our research has other notable limitations. First, compared with definition of myocardial injury using only cardiac troponin values, the definition of myocardial injury combined both cardiac troponin values and ECG findings in the study, which may underestimate the incidence of myocardial injury. Secondly lies in the relatively small sample size and the skewed distribution of some of the study data, which impacts the analysis of relevant indicators. In addition, this is a retrospective study, not prospective, the prognosis of these patients cannot be monitored over time. Furthermore, this was a study conducted in a tertiary liver disease specialized hospital rather than a general hospital, thus the results of the study may differ from those in the general population. Finally, this is a Chinese study and may not be suitable for other races. Therefore, the conclusions of this study need to be confirmed by further large-scaled prospective studies.

## Data Availability

The data supporting the findings of this study are available from the corresponding author upon reasonable request.

## Abbreviations

EGVB: Esophagogastric variceal bleeding
AUGIB: Acute upper gastrointestinal bleeding
AMI: Acute myocardial infarction
RAAS: Renin–angiotensin–aldosterone system
SNS: Sympathetic nervous system
Hb: Hemoglobin
Hct: Red blood cell specific volume
PLT: Platelet
BUN: Urea nitrogen
Cr: Creatinine
ALT: Glutamate pyruvate aminotransferase
AST: Glutathione transaminase
TBIL: Total bilirubin
INR: International normalized ratio
PT: Prothrombin time
APTT: Activated partial thromboplastin time
ICU: Intensive Care Unit
